# Correction: Mechanochemical generation of acid-degradable poly(enol ether)s

**DOI:** 10.1039/d1sc90053f

**Published:** 2021-03-23

**Authors:** Jinghui Yang, Yan Xia

**Affiliations:** Department of Chemistry, Stanford University Stanford California 94305 USA yanx@stanford.edu

## Abstract

Correction for ‘Mechanochemical generation of acid-degradable poly(enol ether)s’ by Jinghui Yang *et al.*, *Chem. Sci.*, 2021, DOI: 10.1039/d1sc00001b.

The authors regret an error in Fig. 3, where Fig. 3(c) and (e) were incorrectly labelled as Fig. 3(e) and (c) respectively. The corrected figure is shown below.

**Fig. 3:**
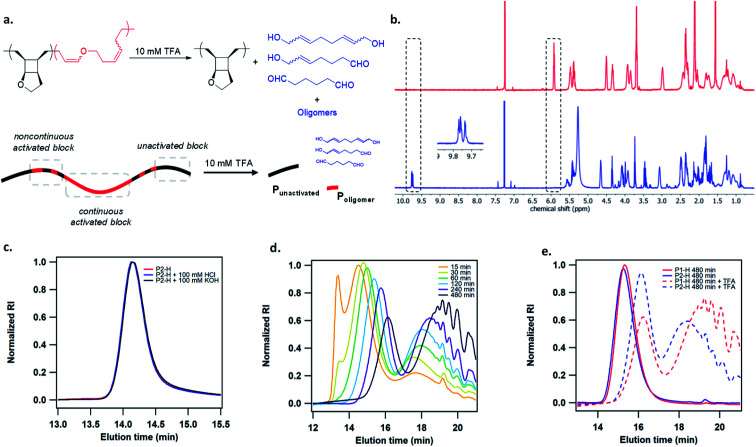
(a) Acid degradation of mechanically activated polymer. (b) ^1^H NMR spectra of mechanically activated poly(**1**) before (top, red trace) and after (bottom, blue trace) 10 mM TFA treatment. (c) GPC traces of hydrogenated poly(**1**) showing resistance to acidic and basic hydrolysis. (d) GPC traces of activated **P1-H** at different times after 10 mM TFA treatment. (e) Comparison of GPC traces of **P1-H** and **P2-H** after 480 min of sonication and 10 mM TFA treatment.

The Royal Society of Chemistry apologises for these errors and any consequent inconvenience to authors and readers.

